# Focus groups inform a mobile health intervention to promote adherence to a Mediterranean diet and engagement in physical activity among people living with HIV

**DOI:** 10.1186/s12889-018-6386-5

**Published:** 2019-01-22

**Authors:** Brook L. Henry, Elizabeth Quintana, David J. Moore, John Garcia, Jessica L. Montoya

**Affiliations:** 1 School of Medicine, Department of Psychiatry, University of California, San Diego, La Jolla, CA USA; 2 Cancer Center, University of California, San Diego, La Jolla, CA USA

**Keywords:** Focus groups, Mediterranean diet, HIV, mHealth, Neurocognition

## Abstract

**Background:**

A personalized mobile health intervention (iSTEP) aims to promote a Mediterranean diet and increase physical activity, thereby improving neurocognitive functioning among persons living with HIV (PLWH). This article describes a qualitative study conducted to develop iSTEP for PLWH, including assessment of diet habits and preferences for tracking physical activity.

**Method:**

Two focus groups, with seven and 13 PLWH respectively, discussed barriers and facilitators of a healthy diet and provided feedback to refine iSTEP components, including the feasibility of using a Fitbit and the content of text messages designed to encourage improved diet and physical activity.

**Results:**

Focus group discussions revealed several healthy diet barriers, including housing instability, time required for food preparation, cost of healthy food, depression, gastrointestinal issues, physical disability, and changes in appetite since HIV diagnosis. Participant-identified healthy diet facilitators included affordable price options for healthy food, a structured food plan, desire to modify appearance or weight, access to medical professionals, motivation for disease prevention, and social support. Participants endorsed wearing a Fitbit on the wrist and receiving text messages as useful methods to monitor and encourage a better diet and greater physical activity.

**Conclusions:**

These findings assisted the expansion of a mobile health intervention that promotes health behaviors in order to improve neurocognitive outcomes among PLWH.

**Trial registration:**

NCT03123731, prospectively registered on ClinicalTrials.gov, April 21, 2017.

## Background

Up to 50% of people living with HIV (PLWH) manifest some degree of neurocognitive impairment, even with effective antiretroviral therapy (ART) [1]. HIV-associated neurocognitive disorders (HAND) may consist of deficits in executive function, attention, and memory, and are implicated in impairments in everyday functioning and poorer quality of life among PLWH [2–4]. Thus, HAND represents a significant public health concern [1], for which there is currently no evidence-based interventions. Among existing lifestyle modification interventions in the general population, promotion of a healthy diet and physical activity (PA) may benefit neurocognitive outcomes among PLWH.

In the natural course of untreated HIV, progressive weight and muscle loss were common; however, with use of effective ART, many PLWH experienced improvements in nutritional status and weight gain [5]. Long-term ART, particularly use of protease inhibitors, may contribute to abnormal fat redistribution, known as lipodystrophy [6]. Body composition changes (e.g., intra-abdominal fat accumulation [7]) are relevant to the pathogenesis of HAND given the association between abdominal obesity and neurocognitive impairment [8]. Both healthy diet and PA can influence body composition alterations among PLWH [5], but the effectiveness of diet and PA interventions to improve neurocognitive outcomes is limited.

Diet and nutrient deficiencies have been extensively documented for PLWH in low-income countries [9]; however, there is limited research characterizing diet quality among adults living with HIV in the United States (US). One US cohort study of adult PLWH with chronic diarrhea reported that mean intake of fat, saturated fat, and cholesterol was higher than recommended levels set by the National Cholesterol Education Program, whereas mean intake of monounsaturated fat, polyunsaturated fat, and fiber fell below recommended levels [10]. While more nutritional data is needed to better characterize diet among PLWH in the US, the limited evidence suggests diet may be a relevant modifiable lifestyle factor to target in interventions that promote better health outcomes.

Adherence to a Mediterranean-style diet (MedDiet) is positively associated with better global neurocognition [11], as well as a lower risk for Alzheimer’s disease, decreased inflammation, and reduced brain atrophy [12, 13]. Despite this body of evidence supporting the MedDiet as a strategy for promoting better neurocognitive outcomes, few studies have investigated the effectiveness of a MedDiet for HAND. Similarly, observational studies indicate a positive association between PA engagement and neurocognitive function, but PA intervention studies among PLWH have yielded inconsistent effects on neurocognitive outcomes [14]. Thus, an approach that targets both PA and diet may be optimal for addressing neurocognitive deficits observed among PLWH.

iSTEP is a novel and personalized text messaging intervention that is currently being evaluated in terms of its effectiveness at promoting moderate PA and reducing sedentary behavior (SB) in PLWH, which in turn, is hypothesized to benefit neurocognitive outcomes. The conceptualization of iSTEP was informed by previous mobile health interventions that show positive effects on behavior change outcomes, such as promotion of PA engagement [15]. As part of the development of iSTEP, focus groups were conducted to elicit feedback from PLWH regarding strategies to increase PA [16]. During these focus groups, the sample of PLWH expressed a myriad of preferences regarding Short Message Service/Multimedia Message Service (SMS/MMS) delivery and content, indicating the need to adopt a personalized intervention approach (e.g., personalized PA text messages, setting individualized PA goals). Preliminary data from the iSTEP study supports high text message response rates and positive participant feedback [17].

The aim of the parent project is to expand the iSTEP intervention to include adherence to a MedDiet, including walnut consumption, thereby improving neurocognitive outcomes among PLWH with HAND. The purpose of this qualitative study was: 1) to characterize diet habits among PLWH, including barriers and facilitators of a healthy diet, and 2) to inform the development of a mobile health intervention (iSTEP) that addresses the preferences of PLWH. The qualitative analysis of focus group content provides a foundation for interventions aimed at behavioral modification as treatment for HAND.

## Methods

### Participants

Two focus groups were conducted with PLWH at the HIV Neurobehavioral Research Program (HNRP) located at the University of California, San Diego (UCSD) Medical Center; one occurred on November 8, 2016 with seven participants and one on November 10, 2016 with 13 participants, for a total of 20 individuals. Participants were recruited through the HNRP based on recent participation in ongoing studies of HIV infection. They were contacted and screened over the phone by HNRP staff and enrolled using consecutive sampling. During the phone interview, participants were administered a brief questionnaire asking if they needed to improve their diet and engage in more PA. To qualify for inclusion, participants had a confirmed diagnosis of HIV, reported concerns about their diet quality, and expressed interest in improving their eating habits. No other inclusion/exclusion criteria were applied. While we did not require specific PA criteria, all participants in the focus groups also indicated that they wanted to be more physically active. Approximately 40 PLWH were screened for the focus groups; 28 met criteria and were scheduled to participate. Of those, eight individuals did not attend, resulting in a final cohort of 20. Some individuals declined to participate because they did not have the time or were not interested in this topic. The HNRP provided a free taxi service to any person who requested this option to ensure that transportation to the meeting was not a barrier to participation. Study approval was obtained from the local Institutional Review Board. Each participant provided written informed consent for the study and received monetary compensation ($35) and an onsite meal.

### Focus group procedure

Each focus group was 1.5 h in duration. The moderator for both groups, Dr. David Moore, a cisgender male, asked predetermined, open-ended questions about diet and elicited feedback on intervention components. At the beginning of each group, Dr. Moore informed the participants that he is a licensed clinical psychologist and UCSD faculty member who has conducted clinical research with PLWH for 20 years, including moderating approximately 20 focus groups among PLWH and/or those at risk for HIV infection. Dr. Moore was not involved in the clinical care of any of the current focus group participants.

The focus groups were structured to cover specific topics, including: (1) existing participant diet habits, (2) barriers to adopting a healthy diet, (3) facilitators for diet improvement, (4) how to support adherence to a Mediterranean-style diet. Dr. Moore guided the discussion from one planned topic to another during the course of the focus group. Dr. Moore also solicited input on the types of text messages that would increase adherence to a MedDiet and promote PA (i.e., gradual increases in step count). Participants were also briefed on two different types of Fitbits—worn on the wrist or waist—including the accuracy and information either model could generate. Focus group participants were asked to express their preferences and concerns to either type of Fitbit. Further, they were asked whether they preferred their Fitbit step count to be remitted singularly or in relation to the group’s results. Group discussion was audiotaped and subsequently transcribed verbatim with participant names de-identified (using coded labels) for data analysis conducted by the study investigators. The transcripts were not shared with anyone outside of the investigator team.

### Data analysis

Transcripts of the focus groups were independently coded by two investigators based on emergent categories related to diet habits, barriers to a healthy diet, and facilitators of a healthy diet. Codes were finalized by consensus among the study investigators and Principal Investigator. Interrater agreement on coded statements was high, as indicated by a kappa value of 0.85. Data analysis was conducted with MAXQDA Analytics Pro 12.3.1 qualitative data analysis software.

## Results

### Participants

The participants were middle aged (M = 54.5 years, SD = 7.2), predominantly male (80%), either non-Hispanic white (40%), black (40%), or Hispanic (20%), with some college education (M = 14 years, SD = 2.4). The open-ended questions used to guide the focus group discussions elicited a variety of participant responses, and our qualitative analysis uncovered an assortment of main themes. These are outlined in Tables 1, 2, and 3, including representative participant statements.

**Table 1:** Current diet habits of focus group participants

Coded theme	Example of coded text
1. Averse diet choices	● “I don’t drink soda; I just don’t really like soda.” ● “I’ll eat anything except sea food.” ● “I don’t like to eat the crap that’s in the center of the store, like can, frozen, and all that crap.”
2. Preferred diet choices	• “I’ve always made it a point to eat a good breakfast, I learned the values of that many years ago, and I’ve always stuck with that.” • “I mostly eat soups, I find it very healthy for me.” • “I have noticed that I am craving lighter foods, like brown rice, tuna, fruits and vegetables, like my body is craving it more.”
3. Proximity to food stores/restaurants	• “I live right across the street from [a produce store], who has all of these wonderful products.” • “I’ll go to [a food bank] and get food there; go to Food-4-Less and get food there. There’s a dollar store near my house. I also go to Big Lot’s and get like bread and chips. It makes my money stretch that way and get a better deal.” • “I live out in the country, to go to a Jimbo’s or someplace like that is really difficult, all the Stater Brothers, Albertsons, and so I’m kind of stuck with either locally grown stuff because I’m out in the hills, and I have to stock up on sweet potatoes from Costco.”

**Table 2:** Barriers to a healthy diet

Coded theme	Example of coded text
1. Housing instability	“Like for me, when you have to transition, it just rocks your whole world. It affects medication, food, stress level.”
2. Time for food preparation	“You don’t just come home and start cooking. You need to get into that zone. My challenge is cooking meals later. Sometimes meals come in a little later, and that’s when a little extra weight comes on for me because I’m eating later and it’s taking more to metabolize.”
3. Cost of healthy food	“I’m really limited financially, so I’m dependent on [a food bank] delivered meals. You know they have a lot of the stuff that is processed, chicken with stuff around it. I try to scrap off the breading and stuff. If I could afford to I would eat a lot better.”
4. Depression or psychiatric issues	“I think when I got diagnosed, depression had set in, and so with depression they say you lose the appetite a little bit or somewhat. I only eat when I’m hungry.”
5. Gastrointestinal issues	“I used to take a lot of meds and had a lot digestive issues.”
6. Physical disability	“I used to like it, but I’ve had two back surgeries, plates and pins put in my back, and I got used to people bringing me food, like [a food bank] pantry.”
7. Changes in appetite since HIV diagnosis	“I ate more [post-HIV diagnosis].”

**Table 3:** Facilitators of a healthy diet

Coded theme	Example of coded text
1. Affordable price options for healthy food	“You catch sales as well in the middle and end of the month. You just get things a little bit at a time and catch things on sale. Week after week, different things.”
2. Structured food plan	“I kind of grew up cooking, mom went to work and dad went to work, so we had the dinner ready for them. My mom was a horticulturalist so I learned a lot from her.”
3. Desire to modify appearance or weight	“I like to look bigger because I think I look better in terms of appearance. I have more confidence in the way I look, and the way I take care of myself. More confident to assert my ideas, instead of feeling like a proverbial 98-pound weakling. I feel better overall if I’m at a certain weight. Like I’m not sickly.”
4. Access to medical professionals or following a doctor’s recommendation	“I go to the doctor a lot and she tells me what to eat, and she’s usually pretty right on, but I’m always learning, that’s why I’m here tonight. I like to eat.”
5. Motivation for disease prevention	“For me personally, my diet is important to me to eat well and healthy because I know that I’m undetectable and healthy, but I don’t what to have anything else like kidney problems, high blood pressure, or diabetes. I try to keep it easy on the soda. I try to subconsciously not eat too much junk food or sweets because I know it can lead to health issues, and I don’t want any other health issues in my life.”
6. Social support	“Having a friend that’s very healthy and eats right. I look up to that and get lots of suggestions.”

#### Current diet habits of focus group participants

Three categories describe dietary habits: (1) adverse diet choices, (2) preferred diet choices, and (3) proximity to food stores/restaurant (Table 1). Some of the adverse dietary choices included soda, seafood, fast food, fried foods, onions, canned or frozen food, processed or breaded food (e.g., breaded chicken). Preferred diet choices included fruits, vegetables, substituting red meat for poultry, grilled or steamed animal protein, organic food, coconut oil, and brown rice. Some participants reported liking chocolate, junk food, ice cream bars, and sweets. Prioritizing breakfast and having meat as part of dinner were also reported as preferred habits. Proximity to fast food, grocery stores, pantries and community churches were also reported to influence diet habits. Living close to fast food restaurants, living in a rural area, and/or having limited financial means were discussed as factors increasing consumption of fast food, potatoes, and delivered meals. Whereas living closer to, working in, or having regular access to grocery stores, pantries or community churches were identified as factors contributing to consumption of fruit and vegetables.

#### Barriers to a healthy diet

The following seven categories were identified as barriers to a healthy diet: (1) housing instability, (2) time required for food preparation, (3) cost of healthy food, (4) depression and other psychiatric issues, (5) gastrointestinal (GI) issues, (6) physical disability, and (7) changes in appetite since HIV diagnosis (Table 2). Participants reported that homelessness or “couch-surfing” impacted the type of foods they consumed, meal timing, and overall health. A reduced desire to cook after a long day or cooking later in the evening was perceived as a diet barrier, as participants linked late-evening eating with weight gain. Cost-specific barriers to a healthy diet included: (a) higher cost of fruits and vegetables; (b) reliance on meal services that may have less healthy options; (c) difficulty budgeting for a months’ supply of groceries; and (d) reliance on a garden that does not always produce. Depression and other psychiatric issues were identified as having an adverse impact on diet, such that these experiences were linked with decreased appetite or weight gain secondary to psychotropic medications. GI issues secondary to medication or drug use were discussed as inhibiting a healthy diet. Physical disabilities, such as back surgery, were discussed as interfering with mobility and thus increasing reliance on others for food delivery. Finally, participants identified various changes in appetite related to HIV that were perceived as barriers to a healthy diet, such as increased appetite following diagnosis and satiety as a side effect of taking medications.

#### Facilitators of a healthy diet

Six general categories were identified as healthy diet facilitators in the focus groups: (1) affordable price options for healthy food, (2) structured food plan, (3) desire to modify appearance or weight, (4) access to medical professionals or following doctor’s recommendation, (5) motivation for disease prevention, and (6) social support (Table 3). Having access to grocery sales (e.g., manager’s deals) or packaged values were identified as facilitators of a healthy diet. Structured food plans, which included preparing one’s own meals or having someone else do so, were discussed as healthy diet facilitators. Participants also reported feeling motivated to eat more (in the case of perceiving oneself as frail or a “weakling”) or less (in the case of being overweight) to modify appearance or weight. Access and adherence to the diet recommendations of a medical provider was viewed as a healthy diet facilitator, as well as motivation for disease prevention (e.g., prevention of high blood pressure, diabetes, kidney problems, and weak immunity). Lastly, social support was mentioned indirectly as supporting a healthy diet. Participants reported having friends and family who cooked for them, invited them for meals, and offered to educate them on healthy eating practices.

#### Text messaging intervention content to promote adherence to the Mediterranean diet

Participants offered suggestions for text message content intended to support adherence to the MedDiet, including daily walnut intake. Participants recommended that texts include recipes (i.e., a variety of recipes that are healthy, simple, affordable, and low-calorie, as well as recipes incorporating walnuts); seasonal information on fruits and vegetables; ideas of non-perishable snacks; and ideas for recipe substitution. Participants also suggested that texts include facts or statistics as to how the prescribed diet may improve health and alter weight, particularly data specific to PLWH. Some focus group participants were interested in receiving personalized texts according to their dietary preferences and having pictures in place of words when possible.

#### Recommendations for the physical activity component of the intervention

Both groups expressed being amenable to wearing a Fitbit and receiving information on steps, heart rate, active minutes, and sleep. Furthermore, wearing a Fitbit on the wrist was reported as more preferable compared to wearing a device on the waist. Participants reported that receiving texts based on their personal progress would be motivating and reporting liking the idea of gradually increasing their steps over time (or meeting their personal threshold) in small increments each week. At least one participant was interested in receiving texts that suggested ways to get more steps (e.g., walking to get groceries or going for a leisurely walk in the park). Participants also specified that PA-related texts should not imply guilt as a way to motivate exercise and preferred the phrase “physical activity” over “exercise” or “working out.” Participants were also apprehensive about group-based interventions due to concerns about between-person competitiveness, differences in level of activity, and issues of confidentiality. Lastly, some participants mentioned the potential utility of receiving a monetary incentive to promote PA engagement, whereas others considered improvement of health and wellbeing as intrinsically motivating.

## Discussion

Focus group discussion yielded identification of prominent healthy diet barriers and facilitators among PLWH. Barriers to a healthy diet included factors observed among the general population (e.g., time demand of food preparation, depression and other psychiatric issues), as well as factors specific to PLWH (e.g., changes in appetite related to HIV and/or ART). Healthy diet facilitators included environmental factors (e.g., access to medical professionals for diet recommendations, affordable price options) and personal factors (e.g., having a structured food plan, desiring to modify appearance or weight). Additionally, focus group discussion served as an avenue to obtain feedback on intervention components (e.g., text content and preferences regarding Fitbit types) that helped inform the expansion of a novel mobile health intervention to promote adherence to a MedDiet, engagement in PA, and reduction of SB among PLWH with HAND. Importantly, focus group discussion highlighted the importance of patient-centered intervention content to promote acceptability of the iSTEP intervention.

Chronic inflammation is implicated in the pathogenesis of HIV-associated neurocognitive impairment across the age span of PLWH [8, 18–23]. The etiology of metabolic disturbance in HIV is likely multi-factorial and includes diets high in cholesterol, saturated and trans fats [24, 25]. Current international guidelines for the treatment of HIV-related dyslipidemia recommend dietary intervention as a first-line treatment [26]. According to one review of dietary interventions in PLWH, administration of omega-3 supplements was successful at lowering triglycerides, but did not have an effect on high-density lipoprotein (HDL) cholesterol, suggesting that a dietary approach that focuses on whole foods might be the most optimal method [27]. Some nutrition studies have promoted polyunsaturated fatty acids (PUFA) and omega-3 PUFA (n-3 PUFA), which are emphasized in the MedDiet [28]. PUFA and n-3 PUFA are present in foods such as walnuts and may benefit brain health and function by reducing oxidative stress and inflammation, maintaining neuronal membrane integrity, and attenuating protein aggregation implicated in neurodegenerative diseases and age-related neurocognitive decline [28]. Adoption of the MedDiet provided reduction in cardiovascular disease events, decreased markers of oxidative stress, and improved neurocognition compared to control or a low-fat diet condition in a large 12-month study [29–34]. Adherence to a MedDiet is associated with improved metabolic function (i.e., lower insulin resistance and higher high-density lipoprotein cholesterol) in PLWH [35]; however, whether improvement in metabolic profile leads to downstream neurocognitive improvement has not been evaluated. Thus, the aim of the parent intervention study is to examine the effect of a MedDiet, including walnut consumption, on cardiovascular risk and neurocognitive outcomes among PLWH.

Many dietary interventions to date have provided a one-size-fits-all approach that may not adequately target barriers and facilitators of a healthy diet at an individual level [36]. Thus, for the expansion of the iSTEP intervention, which includes a focus on promoting adherence to a MedDiet, focus groups were conducted to better understand unique experiences related to dietary behaviors. Many of the participant-identified barriers to a healthy diet are consistent with those described previously among the general population [37–40]. For example, in a sample of persons aged 65 years and older, a common barrier to healthy eating was price [37], which is consistent with a cross-sectional study of middle-aged African American adults who reported that the high price of healthy foods was the biggest barrier to healthy eating [41]. In a study in which participants were provided with a healthy diet (based on dietary reference values for energy and nutrient requirements) for three consecutive days and were then asked during a semi-structured interview about perceived barriers to continuing eating a healthy diet, the most commonly endorsed barriers were competing demands (e.g., work pattern and social activities), a mismatch between a healthy diet and current diet habits of family or household members, not liking the taste of certain foods, and costs associated with a healthier diet [39]. A small body of literature focusing on adoption or adherence to a MedDiet in non-Mediterranean areas have identified barriers such as difficulty changing food habits, limited access to certain food items, increase in food costs, and time constraints [42–44]. Although many of the barriers identified in our study have been previously reported (e.g., time demands for food preparation and issues of cost), our study also identified barriers that may be specific or more pronounced for PLWH, such as a gastrointestinal issues, physical disability, and changes in appetite since HIV diagnosis. Together, the results of our qualitative analysis and previous studies identifying factors related to a healthy diet indicate that improvement of diet may need to address both personal and social barriers.

Our focus group discussions also focused on facilitators of a healthy diet, given that many PLWH in our sample may already adhere to components of a healthy diet currently or in the past. Focus group members discussed the helpfulness of social and environment factors (e.g., affordable price options for healthy foods, access to medical professionals who provide dietary recommendations), as well as personal factors (e.g., desire to modify appearance or weight and motivation for disease prevention). In a previous study focused on factors related to healthy eating, personal factors were emphasized, such as a desire to stay healthy, prevent disease, and promote a high quality of life [37]. In another study utilizing latent class cluster analysis to identify clusters of participants with similar influences on health behaviors, participants in the “low barriers” cluster had lower perceived barriers, higher self-efficacy, higher social support, and more resources for healthy diet compared to participants in the “high barriers” cluster [45]. Thus, interventions aimed at promoting adherence to a healthy diet may identify and provide additional support to participants with fewer personal and social factors supporting healthy dietary behaviors.

The initial aims of iSTEP intervention were to increase moderate PA and improve neurocognition in PLWH with HAND. In the current iteration of iSTEP, the objective is to expand the SMS intervention to also support adherence to a MedDiet. A systematic review and meta-analysis indicated that mobile health interventions promote small decreases in SB and have positive, small-to-moderate effects for PA and walking outcomes [46]. SMS has been used in studies involving PLWH to assess a variety of health indicators and behaviors, such as medication adherence, food insecurity, and alcohol use [47]. SMS interventions designed for PLWH have aimed to promote a variety of health behaviors, particularly medication adherence [48, 49]. In a structured pharmaceutical care intervention (INFAMERICA study), PLWH who were sent periodic text messages about health promotion (e.g., recommendations for improving diet, exercise, and smoking cessation) showed reductions in cardiovascular risk index scores, higher rates of tobacco cessation, and improved medication adherence [50]. The results of the INFAMERICA study provide initial support of the efficacy of SMS interventions to improve health outcomes among PLWH; however, additional research is needed to establish the efficacy of SMS interventions to simultaneously promote a healthy diet and PA among PLWH.

A review of PA and diet mHealth interventions conducted in developing countries for individuals with non-communicable diseases (diabetes and hypertension) reported that the majority of studies significantly improved eating habits [51]. Many of these interventions incorporated text messages that included reminders and instructions to follow a diet regimen and information about the benefits of healthy eating. It is worth noting, though, that not all diet mHealth interventions are successful. McCarroll and colleagues observed that approximately half of mHealth RCTs focused specifically on improving diet demonstrated positive outcomes such as increased vegetable consumption or weight loss [52]. The most successful protocols involved self-monitoring of diet consumption via texts or a cell phone app and feedback that was directly tailored to the self-monitored measures, indicating the importance of integrating these two components.

Similar to the INFAMERICA study, the current iteration of iSTEP aims to intervene on multiple health behaviors (i.e., increase PA, reduce SB, and promote adherence to a MedDiet). Given the multitude of factors impacting engagement in health behaviors, iSTEP is designed to deliver daily personalized and interactive texts in lieu of offering a one-size-fits-all approach. For example, the iSTEP intervention will provide texts with recommendations for healthy food options and recipes based on individual preferences, a strategy endorsed by the focus group participants. iSTEP also provides weekly feedback to the participants on their PA/SB and self-monitored MedDiet adherence, including fruit, vegetable, fish, and legume intake. This intervention is therefore designed to integrate behavioral modification techniques previously shown to be useful, such as adopting a personalized approach with each participant, applying weekly diet self-monitoring, and structuring participant feedback based on changes in their responses over the course of the study. Identifying the relative efficacy of each of these individualized components will be an important step toward improving and refining future patient-centered mobile health interventions.

The current study has several limitations. Our study was a relatively small sample size for qualitative research. Consequently, saturation of categories may not have been reached. All participants had previously participated in other studies at the HNRP, a potential source of sample bias. Although our focus group members were amendable to a mobile health intervention to promote a MedDiet and engagement in PA, the parent prospective clinical trial is needed to determine acceptability of the intervention. Despite these limitations, the perspectives of this sample of PLWH remains important for identifying barriers and facilitators to a healthy diet and obtaining input on a mobile health intervention aimed at promoting adherence to a MedDiet.

## Conclusions

This qualitative study demonstrates the utility of focus groups to better understand patient perspectives of the barriers and facilitators to health behaviors, such as adherence to a healthy diet. Participant-identified barriers to a healthy diet included both general and HIV-specific factors. Development of patient-centered content that addresses the myriad of influences impacting engagement in health behaviors may be warranted to increase the acceptability of interventions. The aim of the parent intervention study is to promote a MedDiet, increase PA, and reduce sedentary behavior, thereby improving neurocognitive function. Findings from the parent RCT will lay the groundwork for development of novel large-scale mobile health inventions to prevent or remediate HAND, which remains a significant public health concern.

## Abbreviations

ART: Antiretroviral medication
HAND: HIV-associated neurocognitive disorders
HNRP: HIV Neurobehavioral Research Program
MedDiet: Mediterranean diet
PA: Physical activity
PLWH: People who live with HIV
PUFA: Polyunsaturated fatty acids
SB: Sedentary behavior
SMS/MMS: Short message service/Multimedia message service
